# Organized Fibrous and Calcified Thrombus of the Medial Canthus Mimicking a Dermoid Cyst: A Clinicoradiological and Histopathological Correlation

**DOI:** 10.7759/cureus.117293

**Published:** 2026-10-01

**Authors:** Houda Safwate, Hala Ait Amar, Yacout Hazzaz, Mehdi Khamaily, Mohamed Elbelhadji

**Affiliations:** 1 Department of Ophthalmology, Faculty of Medicine, Mohammed VI University of Health Sciences (UM6SS), Casablanca, MAR; 2 Department of Ophthalmology, Mohammed VI International University Hospital, Casablanca, MAR

**Keywords:** dermoid cyst, dystrophic calcification, histopathology, medial canthus, organized thrombus, vascular lesion

## Abstract

Medial canthal masses encompass a broad range of lacrimal, congenital, inflammatory, vascular, and neoplastic lesions. Their clinical and radiological features may overlap, making an accurate preoperative diagnosis challenging. We report the case of a 31-year-old woman presenting with a painless, slowly enlarging right medial canthal mass with a four-year history. Examination revealed a firm, non-tender, and non-compressible lesion without inflammatory signs, epiphora, proptosis, diplopia, or ocular motility restriction. Lacrimal irrigation confirmed a patent drainage system. Ultrasonography demonstrated an 11 × 10 mm lesion containing a central hyperechoic focus with posterior acoustic shadowing, consistent with calcification. Computed tomography dacryocystography showed a well-circumscribed medial canthal mass with central calcification and a patent ipsilateral lacrimal drainage system. A dermoid cyst was suspected preoperatively. Complete excision was performed through an external medial canthal approach. Histopathological examination demonstrated a large-caliber vascular structure with a lumen largely obliterated by an organized thrombus composed of fibrin and entrapped red blood cells, with dense fibrosis, cholesterol clefts, and dystrophic calcification. These findings established the diagnosis of an organized fibrous and calcified thrombus. The postoperative course was uneventful, with satisfactory healing and no clinically evident recurrence at the two-month follow-up. This case illustrates how advanced organization, fibrosis, and calcification can obscure the vascular nature of a lesion and closely mimic a dermoid cyst. In this case, histopathological examination was decisive in establishing the definitive diagnosis.

## Introduction

The medial canthal region is anatomically complex, containing the lacrimal drainage system, angular vessels, periosteum, and adjacent orbital soft tissues. Consequently, lesions arising in this location encompass a broad spectrum of congenital, inflammatory, vascular, and neoplastic disorders. Because many of these entities may present as medial canthal masses with overlapping clinical and radiological features, establishing an accurate preoperative diagnosis can be challenging [1].

Among benign lesions considered in the differential diagnosis, dermoid cysts typically present as painless, well-circumscribed periocular masses. Although they are more frequently diagnosed during childhood, they may also present in adulthood [2-4]. On computed tomography (CT), dermoid cysts are generally well circumscribed and may demonstrate variable internal characteristics, calcification, or adjacent bony changes [2-4]. However, these imaging findings are not specific and may overlap with those of other pathological processes involving the medial canthal and orbital regions [1,4].

Vascular lesions represent a less common but important component of the differential diagnosis. Their clinical appearance depends on their vascular characteristics and hemodynamics. Slow-flow venous lesions may exhibit compressibility, positional enlargement, bluish discoloration, or enlargement with increased venous pressure [5-7]. Thrombosis may alter the clinical and imaging appearance of vascular lesions, while calcification or phlebolith formation may occur in chronic venous lesions [5-7]. With chronicity, a thrombus may undergo organization with progressive fibrous remodeling and subsequent dystrophic calcification. These changes may obscure the vascular nature of the lesion and produce a firm, calcified mass mimicking a nonvascular lesion.

Organized thrombotic lesions are an uncommon consideration in the differential diagnosis of medial canthal masses. Disorders involving the angular venous system, including thrombosis and venous abnormalities, may present as medial canthal lesions and create diagnostic confusion with more common periocular or lacrimal disorders [7]. We describe an organized fibrous and calcified thrombus of the medial canthus that clinically and radiologically mimicked a dermoid cyst. Unlike previously reported angular vein disorders, no direct continuity with the angular vein or underlying venous malformation was demonstrated in the present case. This case illustrates how advanced thrombotic organization and calcification may obscure the vascular nature of a lesion and highlights the importance of clinicoradiological-pathological correlation in establishing the definitive diagnosis.

## Case presentation

A 31-year-old woman was referred to our ophthalmology department for evaluation of a painless, slowly enlarging mass involving the right medial canthal region. The lesion had first been noticed approximately four years earlier and had gradually increased in size. There was no history of facial or orbital trauma, previous surgery, acute inflammatory episodes, or rapid enlargement. The patient denied epiphora, recurrent dacryocystitis, purulent discharge, diplopia, or visual disturbances.

On ophthalmologic examination, best-corrected visual acuity was 20/20 in both eyes. Extraocular movements were full, with no restriction of ocular motility or diplopia. There was no proptosis, and intraocular pressure was within normal limits bilaterally. Slit-lamp examination of the anterior segment and dilated fundus examination were unremarkable.

External examination revealed a well-circumscribed subcutaneous mass measuring approximately 10 mm in the right medial canthal region (Figure 1). On palpation, the lesion was firm, non-tender, and non-compressible. There was no overlying erythema, skin discoloration, ulceration, or evidence of local inflammation. No change in size was observed during the Valsalva maneuver. Thus, no typical clinical signs suggestive of a vascular lesion were present.

**Figure 1:**
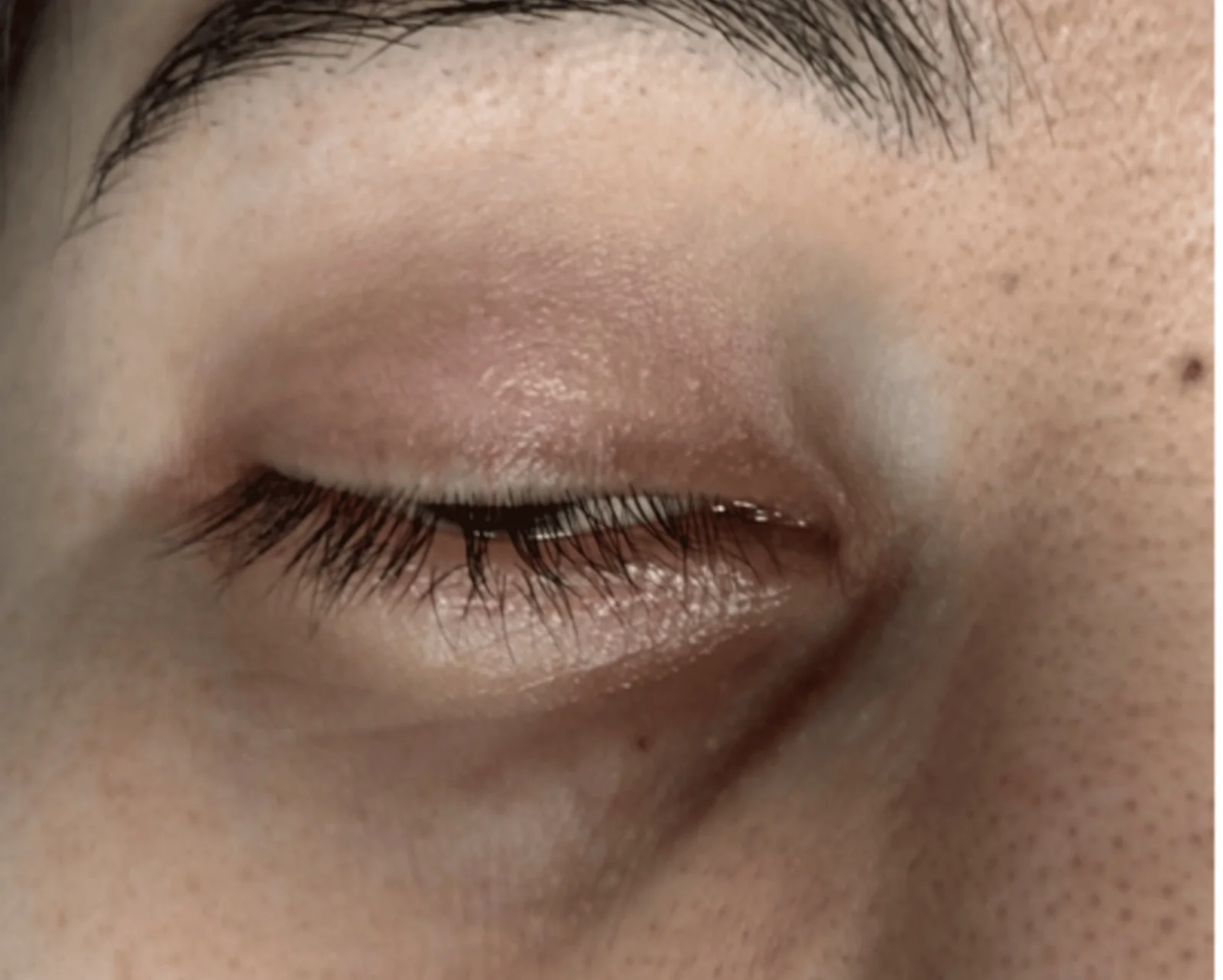
Preoperative clinical appearance of the right medial canthal mass. Preoperative photograph with the right eye closed demonstrating a localized, well-circumscribed swelling of the medial canthal region without overlying skin discoloration or inflammatory changes.

Given the proximity of the lesion to the lacrimal drainage system, lacrimal irrigation was performed and demonstrated a patent drainage system without reflux or evidence of obstruction.

Orbital ultrasonography demonstrated a well-defined 11 × 10 mm lesion containing a central hyperechoic focus with posterior acoustic shadowing, consistent with calcified material. Color Doppler imaging was not performed.

Computed tomography dacryocystography demonstrated a well-circumscribed, round soft-tissue lesion measuring 11 × 10 mm in the right medial canthal region, with regular margins and a central calcified focus (Figure 2). The ipsilateral lacrimal drainage system was adequately opacified. The superior and inferior canaliculi were patent and of normal caliber, without evidence of stenosis; the lacrimal sac was normal in size and morphology, and the nasolacrimal duct was regular and opacified throughout its course. Although the lesion was located adjacent to the lacrimal drainage system, no direct continuity with the angular vein or another specific vascular structure could be established on the available imaging. The radiological findings were considered suggestive of a right medial canthal dermoid cyst, with no evidence of lacrimal drainage obstruction.

**Figure 2:**
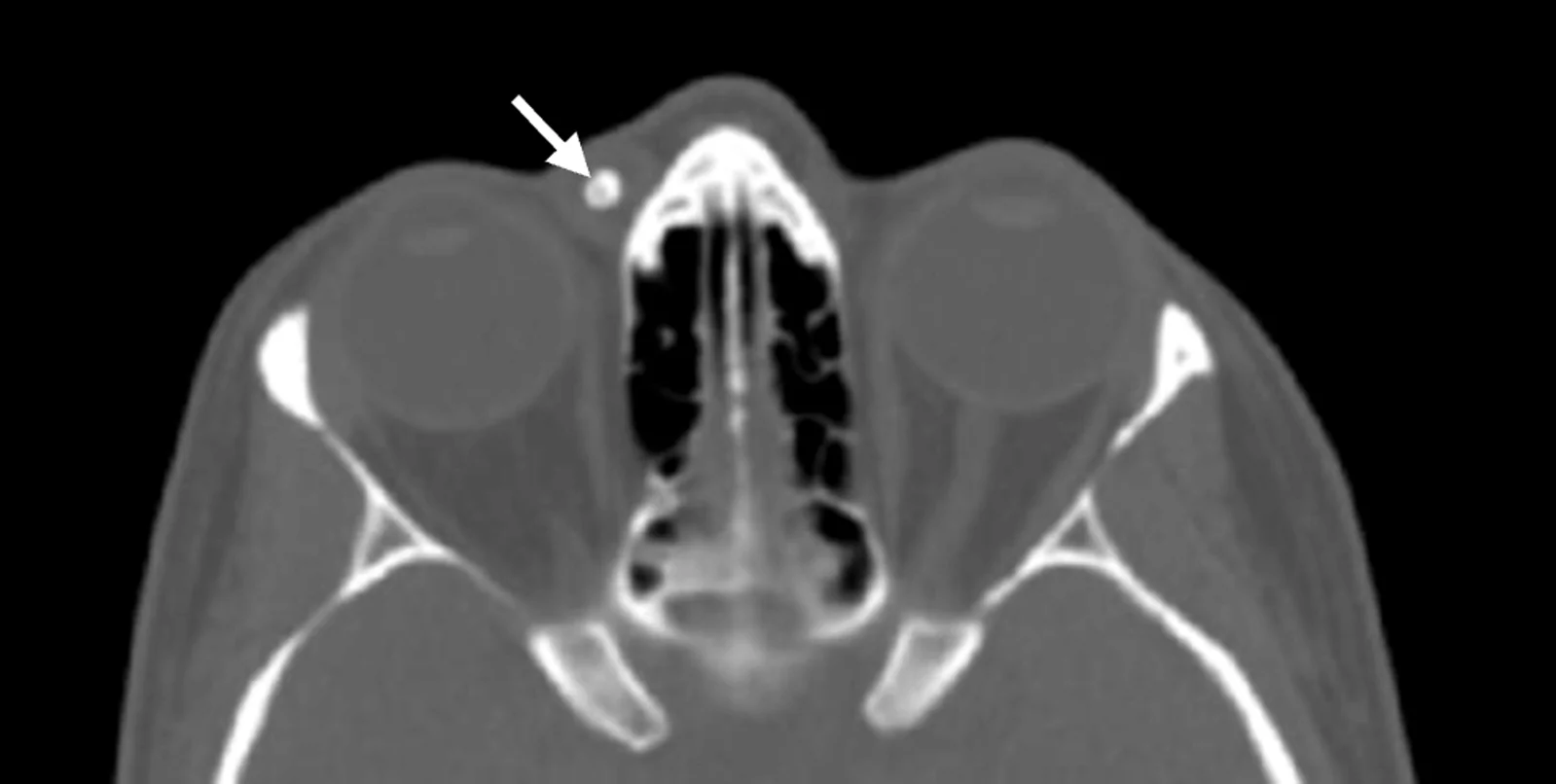
Computed tomography appearance of the right medial canthal lesion. Axial computed tomography image demonstrating a well-circumscribed right medial canthal lesion with a prominent central calcified focus (arrow).

The patient underwent complete surgical excision through an external medial canthal approach under general anesthesia. A small skin incision was made approximately 10 mm from the medial canthus along a natural skin crease, followed by careful blunt and sharp dissection. Intraoperatively, the lesion appeared as a well-circumscribed, firm, yellowish-white mass that could be readily separated from the surrounding tissues (Figure 3A). It was excised en bloc without rupture. No communication with the lacrimal drainage system was identified intraoperatively. On gross pathological examination, the excised specimen measured 0.8 cm in greatest dimension; this ex vivo measurement was reported separately from the approximately 10-mm clinical estimate and the 11 × 10-mm radiological dimensions obtained preoperatively (Figure 3B).

**Figure 3:**
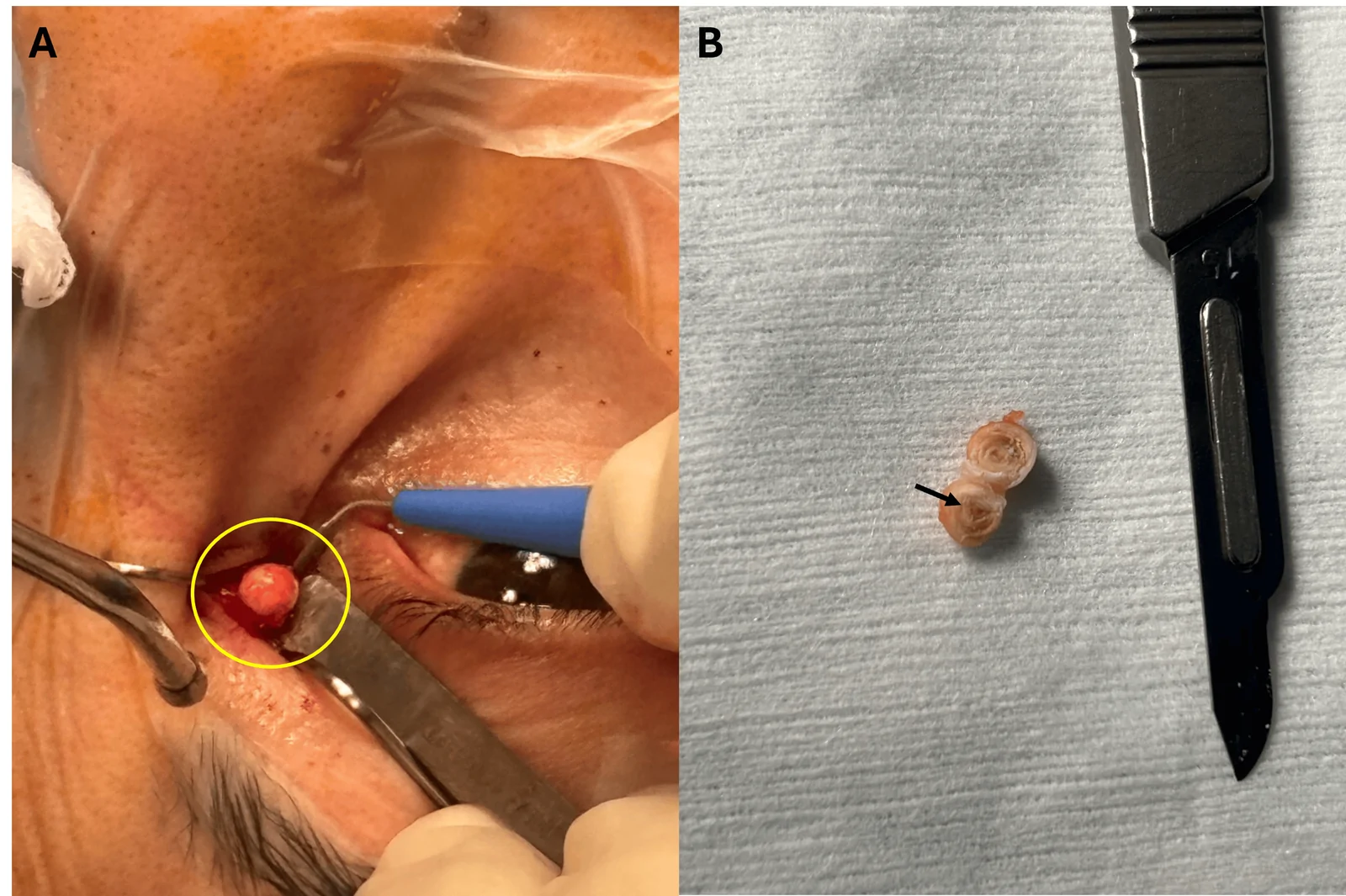
Intraoperative and gross appearance of the right medial canthal lesion. (A) Intraoperative photograph showing the well-circumscribed lesion exposed during surgical dissection through an external medial canthal approach (yellow circle). (B) Gross appearance of the sectioned surgical specimen demonstrating a well-defined lesion with a firm, concentrically organized internal component (black arrow).

Histopathological examination demonstrated a large-caliber vascular structure with a lumen largely obliterated by an organized thrombus composed of fibrin and entrapped red blood cells. The thrombus showed dense fibrous remodeling, cholesterol clefts, and extensive dystrophic calcification, particularly adjacent to the vascular wall (Figure 4). No cytological atypia or evidence of neoplastic proliferation was identified. These findings established the diagnosis of an organized fibrous and calcified thrombus; however, the precise vessel of origin and the presence of an underlying vascular malformation could not be established.

**Figure 4:**
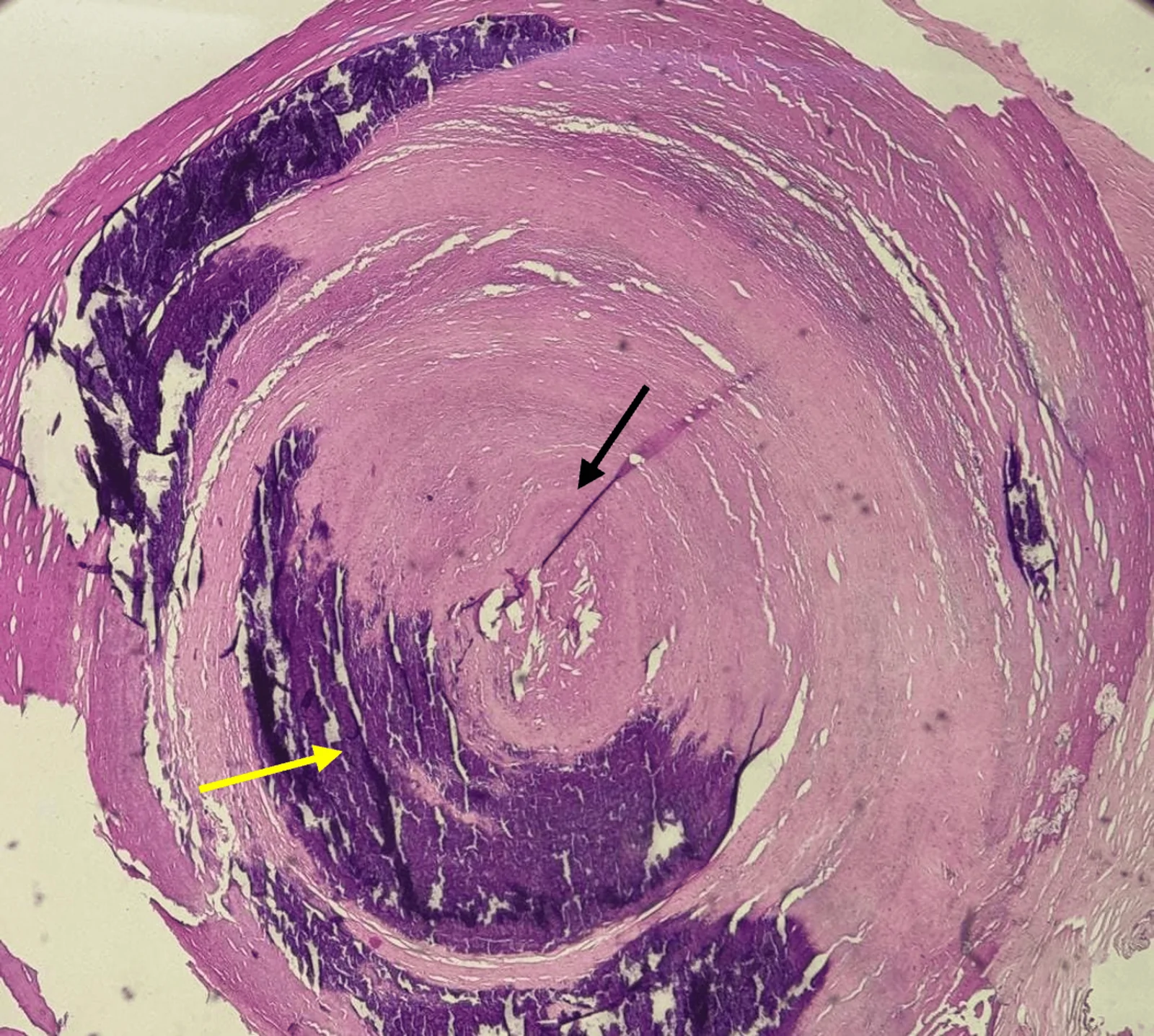
Histopathological features of the organized fibrous and calcified thrombus. Histopathological section of a large-caliber vessel showing a vascular lumen largely obliterated by an organized thrombus (black arrow), with dense fibrous remodeling and extensive foci of dystrophic calcification adjacent to the vascular wall (yellow arrow). No cytological atypia or evidence of neoplastic proliferation is identified (hematoxylin-eosin-saffron (HES), original magnification ×40).

The postoperative course was uneventful. At the two-month follow-up, the surgical wound had healed satisfactorily, with no clinically evident recurrence, lacrimal dysfunction, or ocular complications.

## Discussion

Medial canthal masses represent a diagnostic challenge because lesions arising from the lacrimal drainage system, periocular soft tissues, vascular structures, and adjacent orbit may share similar clinical and radiological features [1]. The differential diagnosis includes congenital cystic lesions, lacrimal drainage disorders, inflammatory conditions, vascular abnormalities, and neoplasms. Vascular disorders of the angular venous system are particularly relevant because they may present as medial canthal swellings and mimic lacrimal or other periocular lesions. Custer et al. described a spectrum of angular vein disorders, including varices, thrombosis, venous malformations, thrombophlebitis, and intravascular tumors, illustrating the diversity of vascular processes that may occur in this anatomical region [7].

In the present case, several features reasonably supported the initial diagnosis of a dermoid cyst. Dermoid cysts typically present as painless, slowly enlarging, well-circumscribed periocular masses and, although more frequently diagnosed in childhood, may also present in adulthood [2-4]. In the series by Dave et al., a painless subcutaneous mass was the most common presentation, including among adult patients [2]. The four-year history of slow progression, firm consistency, well-defined appearance, and central calcification in our patient therefore created a convincing dermoid-like presentation. Furthermore, lacrimal irrigation and CT dacryocystography demonstrated a patent lacrimal drainage system, making an obstructive lacrimal disorder less likely. The radiological interpretation itself favored a medial canthal dermoid cyst.

In this case, the definitive diagnosis was established by histopathological examination. The excised lesion consisted of a large-caliber vascular structure with a lumen largely obliterated by an organized thrombus, associated with dense fibrous remodeling, cholesterol clefts, and extensive dystrophic calcification. These findings are consistent with a chronic thrombotic process with advanced organization, fibrosis, and dystrophic calcification. Importantly, they establish the presence of an organized fibrous and calcified thrombus but do not demonstrate an underlying venous malformation. Therefore, in the absence of histological or intraoperative evidence of abnormal venous channels, the lesion should not be interpreted as a proven venous malformation. Similarly, although calcification within venous thrombi may give rise to phleboliths, the present lesion is more accurately described according to the histopathologically established finding of an organized fibrous and calcified thrombus.

This distinction may also help explain the unusual clinical presentation. Slow-flow venous lesions commonly exhibit features suggesting their vascular nature, including compressibility, positional enlargement, bluish discoloration, or enlargement with increased venous pressure [5,6]. None of these features was present in our patient: the lesion was firm and non-compressible, the overlying skin showed no discoloration, and its size did not change during the Valsalva maneuver. Extensive thrombotic organization, fibrosis, and dystrophic calcification provide a plausible explanation for the loss of recognizable vascular characteristics. However, because the original vascular substrate cannot be established from the available findings, this mechanism should be regarded as a clinicopathological interpretation rather than evidence of a pre-existing vascular malformation.

Imaging further contributed to the diagnostic challenge. Ultrasonography demonstrated a well-defined lesion with a central hyperechoic focus and posterior acoustic shadowing, while CT showed a regular, well-circumscribed 11 × 10 mm medial canthal lesion containing central calcification. Calcification and phlebolith formation can occur in slow-flow venous lesions, whereas CT is particularly useful for detecting mineralization and assessing the anatomical relationships of orbital and periocular masses [5,6,8]. However, calcification is not specific to vascular pathology and may also occur in other lesions, including dermoid cysts [2-4,8]. Thus, in the absence of recognizable vascular features, the presence of calcification alone was insufficient to suggest the final diagnosis.

The medial canthal location also raises the possibility of involvement of the angular venous system. Angular vein disorders may manifest as medial canthal masses, and thrombosis has been documented among the pathological processes affecting this vascular territory [7]. However, no direct continuity between the lesion and the angular vein or another specific vessel was demonstrated intraoperatively or histologically. Assigning a precise vessel of origin would therefore be speculative.

The principal value of this case lies in the clinicoradiological-pathological discrepancy. Clinically, the lesion appeared to be a benign, non-vascular mass; ultrasonography and CT demonstrated a sharply circumscribed calcified lesion, with the radiological interpretation favoring a dermoid cyst; yet histopathological examination revealed a vascular lumen largely replaced by a densely organized and calcified thrombus. This sequence demonstrates how advanced thrombotic remodeling may obscure the vascular nature of a lesion and produce a convincing clinical and radiological mimic.

Several limitations should be acknowledged. Color Doppler ultrasonography and contrast-enhanced magnetic resonance imaging were not performed because a vascular lesion was not suspected preoperatively. These modalities may have provided additional information regarding vascularity, flow characteristics, and the relationship of the lesion to adjacent vascular structures [5,6]. However, very slow flow or extensive thrombosis may reduce detectable vascular signals, and it cannot be assumed retrospectively that either modality would necessarily have established the correct diagnosis [5,6]. Additional histopathological staining, including elastic stains, might have assisted in further characterizing the vascular wall and vessel of origin, but was not performed. Finally, an underlying vascular malformation could not be demonstrated, and postoperative follow-up was limited to two months, allowing only short-term assessment of the surgical outcome. These limitations prevent further characterization of the lesion beyond the histopathologically established diagnosis.

The main clinical lesson is therefore not simply the unusual histopathological finding, but the diagnostic pitfall it represents. A longstanding, firm, non-compressible, calcified medial canthal mass may still represent an organized vascular thrombotic process even when classical vascular signs are absent. In this case, histopathological examination was decisive in establishing the definitive diagnosis and avoiding unsupported assumptions regarding the underlying vascular abnormality.

## Conclusions

An organized fibrous and calcified thrombus may present as a longstanding, firm, calcified medial canthal mass even in the absence of typical clinical features suggestive of a vascular lesion. Advanced thrombotic organization, fibrosis, and dystrophic calcification may obscure the vascular nature of the lesion and closely mimic more common benign entities such as a dermoid cyst, although the underlying vascular origin may remain uncertain. This case highlights the diagnostic limitations of clinical and radiological assessment alone. In this patient, histopathological examination was decisive in establishing the definitive diagnosis.
